# Incidence of and Risk Factors for Pediatric Metachronous Contralateral Inguinal Hernia: Analysis of a 17-Year Nationwide Database in Taiwan

**DOI:** 10.1371/journal.pone.0163278

**Published:** 2016-09-29

**Authors:** Cheng-Hung Lee, Yun Chen, Chi-Fu Cheng, Chao-Lin Yao, Jin-Chia Wu, Wen-Yao Yin, Jian-Han Chen

**Affiliations:** 1 Department of General Surgery, Buddhist Dalin Tzu Chi Hospital, Chia-Yi, Taiwan; 2 Department of Surgery, Far Eastern Memorial Hospital, Pan-Ciao, New Taipei, Taiwan; 3 School of Medicine, Tzu Chi University, Hualien, Taiwan; 4 Department of Chemical Engineering and Materials Science, Yuan Ze University, Chung-Li, Taoyuan City, 32003, Taiwan; 5 Graduate School of Biotechnology and Bioengineering, Yuan Ze University, Chung-Li, Taoyuan City, 32003, Taiwan; Georgia Regents University, UNITED STATES

## Abstract

**Background:**

Previous prospective, retrospective, and meta-analysis studies revealed that the overall incidence of metachronous contralateral inguinal hernia (MCIH) ranges from 5.76% to 7.3%, but long-term follow-up postoperative data are scant. We identified the incidence and risk factors of MCIH in pediatric patients during the follow-up using the Taiwan National Health Insurance Research Database (NHIRD).

**Methods:**

Between 1996/01/01 and 2008/12/31, all pediatric patients with primary unilateral inguinal hernia repair who were born after 1996/01/01 were collected via ICD-9 diagnostic and procedure codes recorded in NHIRD. Patients with another operation during the same admission, complicated hernia, or laparoscopic procedure were excluded. Several reported risk factors, including age, sex, preterm birth, low body weight, and previous ventriculoperitoneal shunt placement, were used for analysis. The primary endpoint was the repairmen of MCIH following the initial surgery. All patients were followed until 2013/12/31 or withdrawal from national health insurance.

**Results:**

A total of 31,100 pediatric patients underwent unilateral inguinal hernia repair, and 111.76 months of median follow-up data were collected. The overall rate of MCIH was 12.3%. Among the 31,100 patients who had the hernia repair, 63.6% had MCIH within 2 years and 91.5% had MCIH within 5 years. After initial surgery, the incidence of MCIH gradually and significantly decreased with age up to approximately 6 years. Multivariable analysis showed that age <4 y and girls were risk factors for subsequent MCIH.

**Conclusions:**

After 17 years of follow-up, the overall MCIH rate was 12.3%, and 91.7% of patients needed repair for MCIH within the first 5 years after initial surgery. Age <4 years and girls were risk factors for MCIH. The contralateral exploration for inguinal hernia should be considered among these patients.

## Introduction

At their first presentation with symptoms of a hernia, most pediatric patients have unilateral disease, but some children develop a hernia on the contralateral side after surgical repair, which is termed metachronous contralateral inguinal hernia (MCIH). The previous literature reviews in 2015 and 2011 revealed MCIH incidences of approximately 6% [1,2]. Initial presentation with a left-sided hernia and age younger than 6 months are risk factors for the development of MCIH [1–3]. Routine exploration of the contralateral, asymptomatic side to prevent the development of MCIH remains debatable because an estimated 18 contralateral explorations are needed to prevent 1 incidence of MCIH [2]. Moreover, unnecessary operations increase the risks for surgical complications [4].

Many prospective, retrospective, and meta-analysis studies have attempted to evaluate the true incidence of pediatric MCIH, but it is still difficult to get long-term follow-up data regarding MCIH after the initial operation. A previous report suggested that only about 6% of the patients were followed up more than 2 years [1].

The National Health Insurance Research Database (NHIRD) is a national resource provided by the National Health Research institutes of Taiwan. This database is a comprehensive and reliable source for the present study because it enrolls about 99% of the population and 97% of medical providers, and it records all medical practices in Taiwan [5]. Once a patient has been admitted to a medical care facility in Taiwan, any medical or surgical treatment he or she receives will be recorded in this database. For our study, almost all patients who received unilateral hernia repair could be followed up by almost every physician in the country until the patient was withdrawn from national health insurance due to death, missing appointments for more than 6 months, or living abroad for more than 6 months.

The purpose of our study was to appraise the incidence of and risk factors for MCIH in pediatric patients by analyzing the data from the Taiwan NHIRD database. These data may provide clinical guidance regarding exploration of the contralateral inguinal area during the patient’s initial hernia repair.

## Materials and Methods

This study is based on the data from the NHIRD, which is managed by National Health Research Institutes (Registered Number NHIRD-103-246). The data was generated by the National Health Insurance Administration and the Ministry of Health and Welfare. This study was approved by the Institutional Review Board of Buddhist Dalin Tzu Chi Hospital (B10304006), who waived the need for inform consent. However, the interpretation and conclusions of this article do not represent the opinion of the government health authorities.

### Study Sample and Identification of Hernia Repair Surgeries

For our analysis, we used data regarding inpatient expenditures by admissions (DD) from the NHIRD database from 1996/01/01 to 2013/12/31. In this database, the diagnosis and procedures associated with each admission were recorded according to the Ninth Revision of the International Classification of Diseases (ICD-9). We selected all patients <18 years old with the discharge diagnosis of hernia (ICD-9-Clinical Modification codes 550.xx to 553.xx) combined with a unilateral inguinal hernia repair (53.00 to 53.05). The National Health Insurance Program started in 1996, and its database held all the medical records of most patients born after 1996/01/01. However, some incomplete information may be recorded in the database for patients born before 1996/01/01, so we excluded this group of patients.

**Fig 1:**
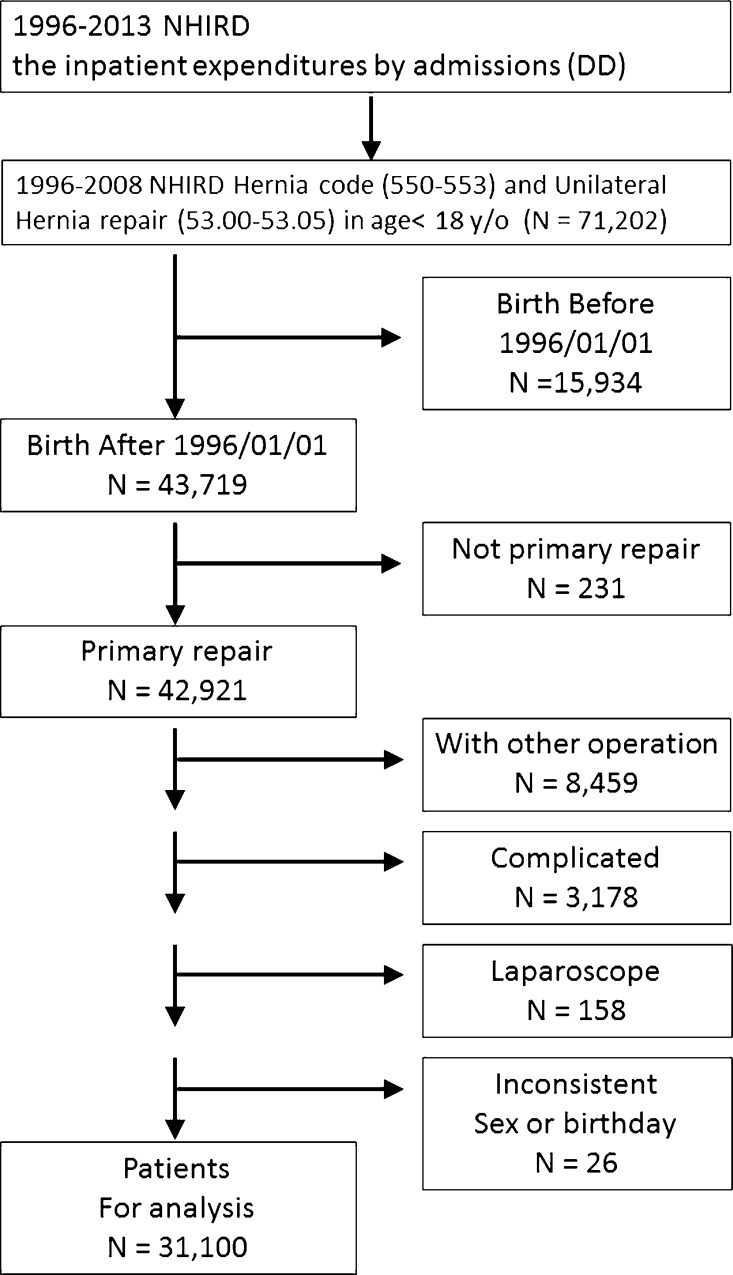
Study flow chart.

We also excluded the following patient groups; patients discharged with a diagnostic code associated with recurrent inguinal hernia (550.01, 550.03, 550.11, 550.13, 550.91, or 550.93), patients received another operation or involved laparoscopic surgery (54.21) during the same admission, or patients treated for a complicated hernia (including 550.0x, 551.x [with gangrene], 550.1x, or 552.x [with obstruction]) [6]. Patients with the inconsistent gender and birthday were also excluded. All patients treated between 1996/01/01 and 2008/12/31 who fulfilled the admission and exclusion criteria were enrolled in this study. The selection algorithm is shown in **Fig 1**.

### Definitions and Risk Factors

The date of admission was defined as the date of hernia repair. To separate any reoperations for surgical complications, each detected MCIH was defined as reoperation if it took place after an interval of more than 30 days following the initial hernia repair. However, we cannot directly detect reoperations in the NHIRD database because the laterality (side of the body affected) of the index hernia repair cannot be identified via ICD-9 code. Therefore, we used the following method to define MCIH:

1. Patients who received a second unilateral hernia repair without ICD-9 diagnostic codes of recurrence of a hernia (550.01, 550.03, 550.11, 550.13, 550.91, or 550.93)
2. Patients who received bilateral inguinal hernia repair (ICD-9 procedure codes 53.10–53.17) after initial unilateral inguinal hernia repair
3. If the second unilateral hernia repair was coded for the recurrence of a hernia, it was defined as a recurrence. However, if we found a third unilateral hernia repair without a recurrence code or specification for bilateral hernia repair, it was defined as MCIH, and so on.

Several factors were taken into consideration in this analysis. Patients were divided into different age groups as follows: 0–6 months, 6 months–1 year, 1–2 years, 2–3 years, 3–4 years, 4–5 years, 5–6 years, and >6 years. Preterm infants (those <28 weeks: ICD-9 codes 765.0x, 765.21–765.24 and those aged 28–36 weeks: 765.1x, 765.25–765.28), those with birth body weight <1500 g (ICD-9 codes: 765.01–765.05, 765.11–765.15), and those who received ventriculoperitoneal shunt placement (ICD-9 surgical procedure code 02.34) [7] were identified by the relevant ICD-9 codes recorded in the NHIRD database from birth to the time of admission.

### Study End Point and Patient Follow-Up

The primary endpoint was MCIH repair. All included patients were followed up until their withdrawal from national health insurance due to death, missing appointments for more than 6 months, living abroad more than 6 months, or the end of the study period, 2013/12/31.

### Statistical Analysis

All data were calculated with MedCalc Statistical Software version 16.2.1, 2016 (MedCalc Software bvba, Ostend, Belgium). The contingency table was created, and the difference of MCIH repair rate between age groups, gender, and comorbidities were identified by the chi-square test. Kaplan–Meier analysis was used to identify the cumulative incidence of MCIH and the trend of repaired MCIH as the following times. Cox regression model was used to evaluate the risk of MICH between different covariant. A value of *p* ≤0.05 was considered statistically significant.

## Results

**Fig 2:**
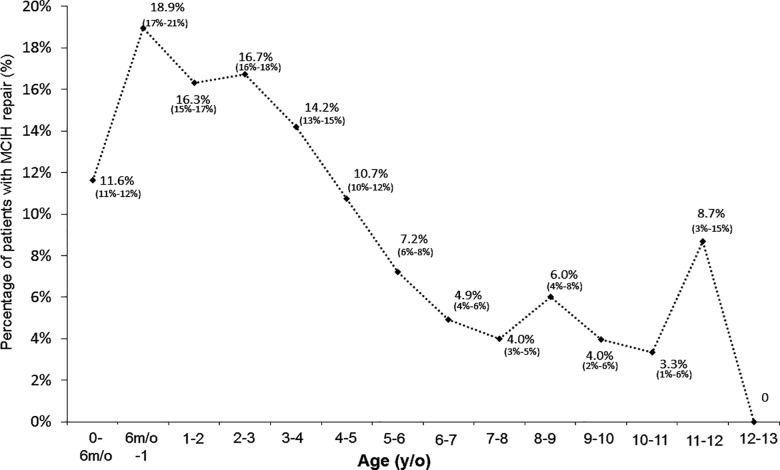
The rates of metachronous contralateral inguinal hernia among different age groups.

**Fig 3:**
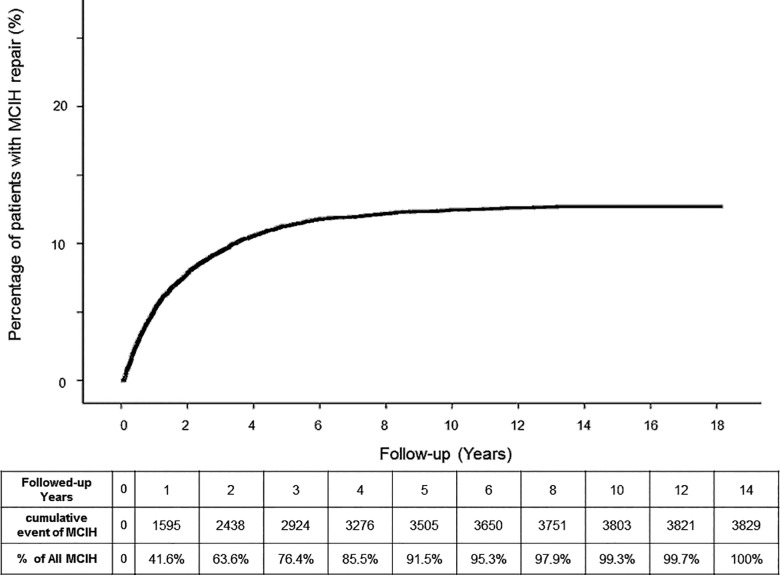
Kaplan–Meier analysis of metachronous contralateral inguinal hernia rates after primary unilateral inguinal hernia repair in pediatric patients.

From 1996/01/01 to 2008/12/31, 31,100 patients fulfilled inclusion criteria and were included for analysis; the median follow-up time was 111.76 months. The overall MCIH rate was 12.3%. The rate of MCIH was significantly different among different age groups: As the age increased, the rate of MCIH decreased (**Fig 2**). Kaplan–Meier analysis demonstrated that the overall cumulative recurrences increased gradually during the follow-up period (**Fig 3**). At the end of the 17-year cohort studied, 12.7% patients had received an operation for MCIH. The median time from primary inguinal hernia repair to MCIH was 15.33 months. The percentages of all MCIH at 1, 2, 3, 4, and 5 years were 41.6%, 63.6%, 76.4%, 85.5%, and 91.5%, respectively.

Table 1 shows the clinical characteristics of the 31,100 pediatric patients included in the study. Patients aged more than 6 years had the lowest rate of MCIH (4.6%). Then the MCIH rate gradually increased as the age decreased. Boys had a higher MCIH rate than did girls, but the difference did not reach statistical significance. Moreover, patients who were born preterm, those who had a birth body weight less than 1500 g, and those with a history of ventriculoperitoneal shunt placement showed no effect on the MCIH rate. All these data are presented in **Table 1**.

**Table 1:** Metachronous contralateral inguinal hernia rates according to different clinical characteristics among 31,100 pediatric patients after primary unilateral inguinal hernia repair.

Clinical Characteristics	Total Patients	MCIH Events	MCIH Rate	*p*
**Age, y**				<0.001
0–6 m/o	5644	656	11.60%	
6 m/o–1 y/o	1854	351	18.90%	
1 y/o–2 y/o	4581	747	16.30%	
2 y/o–3 y/o	4385	733	16.70%	
3 y/o–4 y/o	4015	570	14.20%	
4 y/o–5 y/o	3406	366	10.70%	
5 y/o–6 y/o	2757	199	7.20%	
>6 y/o	4458	207	4.60%	
**Sex**				0.08
Boy	24,731	3086	12.50%	
Girl	6,369	743	11.70%	
**Preterm**				0.804
No	30,968	3,812	12.30%	
Yes	132	17	12.90%	
**Birth BW <1500g**				0.333
No	30,611	3762	12.30%	
Yes	489	67	13.70%	
**VP Shunt**				0.651
No	31,055	3825	12.30%	
Yes	45	4	8.90%	

Abbreviations: BW, body weight; VP shunt, ventriculoperitoneal shunt; m/o, months old; y/o, years old.

### Multivariable Analysis

We used Cox regression analysis to study all variables that had a *p* value <0.2. The factors that contributed to significantly different outcomes were identified from **Table 2** according to multivariable analysis. We used the 4–5-year-old group (MCIH rate: 10.7%) as a baseline. On the other hand, the risk decreased as the ages were increased. Girls had a significantly higher risk for MCIH than did boys (HR = 1.114, 95%CI: 1.027–1.209, *p* = 0.009).

**Table 2:** Risk factors associated with metachronous contralateral inguinal hernia rates among 31,100 pediatric patients after primary unilateral inguinal hernia repair as shown by Cox regression analysis.

Clinical Characteristics	HR	95% CI	*P*
**Age, y**			
0–6 m/o	1.106	(0.973–1.257)	0.125
6 m/o–1 y/o	1.859	(1.605–2.154)	<0.001
1 y/o–2 y/o	1.578	(1.391–1.790)	<0.001
2 y/o–3 y/o	1.634	(1.441–1.854)	<0.001
3 y/o–4 y/o	1.364	(1.196–1.555)	<0.001
4 y/o–5 y/o	1		
5 y/o–6 y/o	0.661	(0.557–0.786)	<0.001
>6 y/o	0.423	(0.357–0.502)	<0.001
**Sex**			
Boy	1		
Girl	1.114	(1.027–1.209)	0.009

Abbreviations: HR, hazard ratio; CI, confidence interval; m/o, months old; y/o, years old.

## Discussion

In this 17-year cohort study, the overall MCIH recurrence rate was 12.3%. After Kaplan–Meier analysis, more than 90% of MCIH developed within the first 5 years after the initial unilateral inguinal hernia repair in pediatric patients. The risk of MCIH gradually increased as the age decreased, and girls was a risk factor for MCIH.

In our study, age was one of the risk factors influencing the incidence of MCIH. Patients with younger age had a higher risk of developing MCIH (data are shown in **Table 2**). In multivariable analysis by Cox regression, we used patients in the 4–5-year-old group as a baseline to evaluate the hazard ratio at different ages. In our study, the incidence of MCIH in this group was about 10%, which suggests that nearly 10 contralateral explorations are needed to prevent 1 MCIH. In the literature, contralateral exploration is not suggested because the reported incidence of MCIH is about 6%–7%, and the number needed to treat (NNT) is around 14–18 [1,2,8]. Although the NNT is increased up to 9 in younger patients, contralateral exploration could be performed after the discussion about the pros and cons with the family [2]. As a result, we decided to use this group of patients as the baseline. The results shown in **Table 2** revealed that the hazard ratio increased with a statistically significant difference as the age decreased.

Moreover, compared to other studies, our study noted a similar trend between lower age and greater numbers of MCIH. However, the incidence of MCIH in patients younger than 3 years old is higher [1,2,9]. On the contrary, patients older than 6 years in this study had a lower incidence of MCIH (4.6%) compared to other data reported in the literature [2]. This was probably caused by the limitations of the database we used. We used only the inpatient expenditures by admissions (DD) from the NHIRD for analysis. We were not able to identify patients with unoperated MCIH or those operated without admission. However, some surgeons in Taiwan perform ambulatory hernia repair operations in older children, so the absolute number of recurrences may be higher than the data suggested. Nevertheless, patients of younger age, especially those younger than 1 year old, are admitted for surgery in most circumstances.

Because of different inclusion criteria and follow-up times, the reported incidence of pediatric MCIH varies greatly [2]. According to previous studies, the overall incidence of MCIH was from 5.76% to 9.2% [1,2,9–12]. In this study, the overall MCIH incidence was 12.3%, which is higher than previously reported, probably because of the longer follow-up period. In this study, we found that 63.6% of patients developed MCIH within 2 years after their first hernia repair, and 76.4% of MCIH developed within 3 years. This result was similar to data reported previously [2]. Even so, Wenk et al in 2015 reported that only 6% of the patients were followed up more than 2 years [1]. To obtain the comprehensive clinical picture after the surgery, we suggest that any future studies in this field should have a longer follow-up period to collect the most events. If not, the MCIH incidence probably will be underestimated. In our study, all patients included in the analysis were followed up at least 5 years, and the median follow-up was 111.76 months. We believe a 5-year follow-up period is sufficient to yield a more precise MCIH rate.

The loss to follow-up can also be a problem in retrospective research that relies on reported follow-up methods such as clinical investigation, phone call, letter, or e-mail [13,14]. In contrast to previous studies, our cohort used NHIRD, which records almost all medical practices in Taiwan and is a national resource provided by the National Health Research Institutes of Taiwan. In the other words, except for those patients who lived abroad or went missing, our study included almost all the repeat hernia repairs after the index repair during the study period. This unique system made the identification of the risk of MCIH more comprehensive and reliable during the long-term follow-up period.

Previously, Chin et al [12] reported that the MCIH rate in Taiwan is 9.2% by using a different data set, the longitudinal health insurance database (LHID 2005), of NHIRD. The reported rate was a little lower than the incidence we report here. This may be related to the different database we used. Chin et al. used LHID 2005, which included for analysis only 1 million beneficiaries randomly drawn from the registry of beneficiaries in 2005. Our study used the inpatient expenditures based on the admissions (DD) database. Admissions details, including diagnosis and procedures recorded by ICD-9-CM code, discharge conditions, and the cost of admission of every civilian, were recorded in this data set. This database identified most patients with unilateral inguinal hernia repair and yielded more precise MCIH rates. However, as we mentioned before, based on the database we used we cannot identify patients with unoperated MCIH or those operated without admission. Even so, we believe that the true MCIH rate in Taiwan is close to the result we report.

### Limitations

There are limitations to this study. First, the inpatient data in this database are administrative in nature, and they rely entirely on accurate ICD-9-CM coding. In this database, we cannot identify the laterality of the index hernia repair, which is reported as an important factor in MCIH [1], because it cannot be distinguished via the ICD-9-CM code. Also, we cannot identify via our database all details of each physician’s notes to clarify the operative method, the severity of the hernia, self-paid materials during the admission, and any other unrecorded factors.

Second, the risk of miscoding exists because the ICD-9 coding is not usually determined by surgeons. Instead, surgeons in Taiwan always gave Health Insurance Surgical Orders from the Taiwan NHI payment system, which directly relates to the revenue for surgeons, immediately after each operation. However, an official comparison table for these different codes was offered by the National Health Insurance Administration Ministry of Health and Welfare. Moreover, most recorded ICD-9 codes in this database were given by professional coders based on the records during admission and were directly related to the hospital’s income. We considered the miscoding of surgical procedure to be limited.

## Conclusion

After analyzing the data from the nationwide database, the results of this 17-year follow-up cohort study show the overall MCIH rate was 12.3%. More than 90% of patients are likely to need repair for MCIH within the first 5 years after primary unilateral inguinal hernia repair. We belive that the longer follow-up period in our study gives more precise data about the MCIH rate. Age younger than 4 years and girls were risk factors for MCIH. In patients with these risk factors, contralateral exploration should be considered and discussed with the family to prevent subsequent secondary MCIH repair.
